# A review on the role of mir-16-5p in the carcinogenesis

**DOI:** 10.1186/s12935-022-02754-0

**Published:** 2022-11-08

**Authors:** Soudeh Ghafouri-Fard, Tayyebeh Khoshbakht, Bashdar Mahmud Hussen, Sara Tharwat Abdullah, Mohammad Taheri, Mohammad Samadian

**Affiliations:** 1 Department of Medical Genetics, School of Medicine, Shahid Beheshti University of Medical Sciences, Tehran, Iran; 2 Men’s Health and Reproductive Health Research Center, Shahid Beheshti University of Medical Sciences, Tehran, Iran; 3 Department of Pharmacognosy, College of Pharmacy, Hawler Medical University, Kurdistan Region, Erbil, Iraq; 4 Center of Research and Strategic Studies, Lebanese French University, Erbil, Kurdistan Region Iraq; 5 Department of Pharmacology and Toxicology, College of Pharmacy, Hawler Medical University, Erbil, Iraq; 6 Institute of Human Genetics, Jena University Hospital, Jena, Germany; 7 Skull Base Research Center, Loghman Hakim Hospital, Shahid Beheshti University of Medical Sciences, Tehran, Iran

**Keywords:** miR-16-5p, Cancer, Biomarker, Expression, Malignancies

## Abstract

miR-16-5p is microRNA with important roles in the development of diverse malignancies including neuroblastoma, osteosarcoma, hepatocellular carcinoma, cervical cancer, breast cancer, brain tumors, gastrointestinal cancers, lung cancer and bladder cancer. This miRNA has 22 nucleotides. hsa-miR-16-5p is produced by *MIR16-1* gene. First evidence for its participation in the carcinogenesis has been obtained by studies reporting deletion and/or down-regulation of these miRNAs in chronic lymphocytic leukemia. Subsequent studies have shown down-regulation of miR-16-5p in a variety of cancer cell lines and clinical samples. Besides, tumor suppressor role of miR-16-5p has been verified in animal models of different types of cancers. Studies in these models have shown that over-expression of this miRNA or modulation of expression of lncRNAs that sponge this miRNA can block carcinogenic processes. In the current review, we summarize function of miR-16-5p in the development and progression of different cancers.

## Introduction

MicroRNAs (miRNAs) are small-sized transcripts that regulate expression of genes at post-transcriptional level through specific targeting of mRNAs. With sizes about 21–25 nucleotides, miRNAs are originated from coding and non-coding transcription units in introns, exons or intergenic areas [1]. They are produced in a multi-step process involving both nuclear and cytoplasmic proteins. They are involved in the carcinogenic process, since they can regulate expression of several oncogenes and tumor suppressor genes as well as activities of cancer-associated pathways [2]. Expression pattern and function of several miRNAs have been assessed in different cancer types. Since these small-sized transcripts are stable in the circulation or other biofluids, they represent potential biomarkers for diagnostic and follow-up purposes [3]. Dysregulation of miRNAs has been correlated with evolution of cancers, hence they are regarded as molecular tools for non-invasive assessment of cancer occurrence and its prognosis [4].

miR-16-5p is an example of this class of transcripts with important roles in the development of diverse malignancies including neuroblastoma, osteosarcoma, hepatocellular carcinoma, cervical cancer, breast cancer, brain tumors, gastrointestinal cancers, lung cancer and bladder cancer. This miRNA has 22 nucleotides and is present in Homo sapiens. Homo sapiens hsa-miR-16-5p is produced by *MIR16-1* gene.

miR-16-1 is allocated at 13q14.3 along with miR-15a. This miRNA cluster is the target of 13q deletions in chronic lymphocytic leukemia (CLL). miRNAs encoded by this locus have tumor suppressor functions. First evidence for its participation in the carcinogenesis has been obtained by studies reporting deletion and/or down-regulation of these miRNAs in (CLL) [5]. The tumor suppressor functions of miR-15a/16 − 1 are exerted through targeting the BCL2 oncogene. Through a high-throughput study in a leukemic cell line model, Colin et al. have found enrichment in AU-rich elements in the elements of the miR-15a/16 − 1 signature [6].

Subsequently, different studies have assessed role of miR-16-5p in the carcinogenesis using in vitro and in vivo techniques. Moreover, expression pattern of miR-16-5p has been evaluated in clinical samples gathered from patients with diverse malignancies. In the current review, we summarize function of miR-16-5p in the development and progression of different cancers using the above-mentioned lines of evidence. The reason for selection of this miRNA in this review article is the important role of this miRNA in the suppression of carcinogenesis, its down-regulation in a variety of solid and hematological malignancies and its potential as an anti-cancer target. The following strategy was used for selection of papers: publication in full-text English language in a peer-reviewed journal and detailed description of conducted methods. In addition, papers should include in vitro functional studies or expression assays in clinical samples.

### Cell line studies

Cell line studies have indicated important roles of miR-16-5p in the carcinogenesis. Moreover, these studies have shown the inhibitory effects of this miRNA on transcription of several genes, particularly a number of known oncogenes. An in vitro study in neuroblastoma has shown interaction between miR-15a, miR‐15b and miR‐16 and MYCN transcript. Based on the results of luciferase reporter assay these miRNAs bind with 3’UTR of MYCN transcript leading to suppression of its expression. Forced up-regulation of these miRNAs has decreased proliferative potential, migratory ability, and invasion of neuroblastoma cells [7]. Another study in neuroblastoma has shown that the oncogenic circular RNA circ-CUX1 enhances tumorigenesis of neuroblastoma and their glycolysis through targeting miR-16-5p. Moreover, miR-16-5p tumor suppressor impact has been partially decreased by transfection of circ-CUX1 overexpressing vectors. DMRT2 has been found to be targeted by miR-16-5p in neuroblastoma cells [8].

**Fig. 1:**
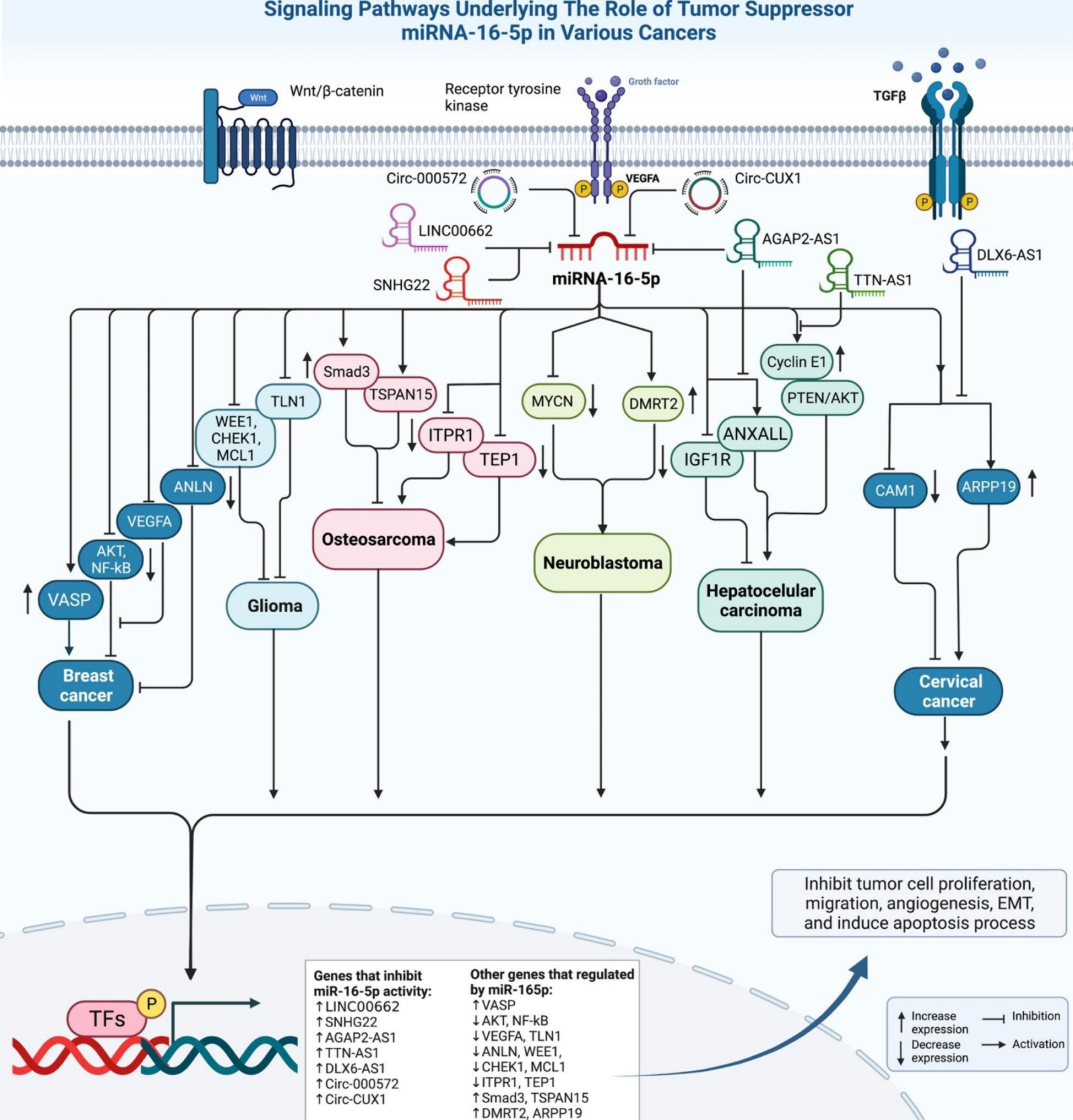
Tumor suppressor role of miR-16-5p in different types of cancer

miR-16-5p has been to be down-regulated in osteosarcoma cell lines compared with control cells, parallel with up-regulation of Smad3. Up-regulation of miR-16-5p has suppressed proliferation, migratory potential and invasive features of osteosarcoma cells and increased the cytotoxic effects of cisplatin on these cells. Moreover, miR-16-5p over-expression has led to reduction of Smad3 expression. Notably, cells harboring Smad3 mutation have not responded to miR-16-5p over-expression, indicating that miR-16-5p suppresses invasive properties of osteosarcoma cells through suppressing expression of Smad3 [9]. miR-16-5p effect in suppression of tetraspanin 15 gene has also been involved in the inhibition of osteosarcoma cells proliferation, migration and invasion [10]. Figure 1 shows tumor suppressor role of miR-16-5p in different types of cancer.

The long non-coding RNA (lncRNA) AGAP2-AS1 which targets miR-16-5p has been shown to be up-regulated in hepatocellular carcinoma cell lines. This lncRNA could promote proliferation, migratory aptitude, invasiveness and epithelial-mesenchymal transition (EMT) of these cells through acting as a sponge for miR-16-5p. ANXA11 has been found as a target of miR-16-5p in hepatocellular carcinoma cells, mediating the impacts of miR-16-5p and AGAP2-AS1 in these cells and enhancing activity of AKT signaling. Notably, hypoxia has been shown to increase levels of AGAP2-AS1 in these cells [11]. Another study has confirmed down-regulation of miR-16-5p in hepatocellular cancer cells. Dual-Luciferase reporter gene assay has validated the regulatory role of miR-16-5p on expression of Insulin like growth factor1 receptor (IGF1R). IGF1R down-regulation has decreased the suppressive role of miR-16- 5p on proliferation ability and metastatic potential of hepatocellular cancer cells [12]. Moreover, down-regulation of miR-16-5p by lncRNA TTN-AS1 has been shown to promote resistance to sorafenib through enhancement of expression of cyclin E1 [13]. Finally, another study in hepatocellular carcinoma has shown that SNHG22 increases tumorigenic ability of cancer cells and their angiogenesis though induction of DNA methylation in miR-16-5p [14].

In cervical cancer cells, miR-16‐5p affects radiosensitivity through regulation of expression of coactivator‐associated arginine methyltransferase 1 [15]. Moreover, it can influence metabolic reprogramming and chemoresistance through regulation of Pyruvate Dehydrogenase Kinase 4 (PDK4) expression [16].

In breast cancer cell, down-regulation of miR-16-5p has been associated with high migratory and proliferative potential of cells, induction of cell cycle progression and reduction of cell apoptosis. miR-16-5p could restrain activity of the Nuclear factor kappa-light-chain-enhancer of activated B cells (NF-κB) pathway and reduce expression of AKT3 gene, thus inhibiting development of breast cancer [17]. miR-16-5p could also suppress proliferation of breast cancer cells through down-regulating expression of ANLN [18]. The inhibitory effect of miR-16 -5p in breast cancer cells proliferation and invasiveness can be mediated through regulation of Vascular Endothelial Growth Factor A (VEGFA) expression [19]. Finally, ATXN8OS has been shown to enhance tamoxifen resistance through sponging miR-16-5p [20].

Moreover, miR-16‐5p has been shown to be commonly down‐regulated in astrocytic gliomas. This miRNA could regulate proliferation and apoptosis of these cells as well as effect of cytotoxic agents on these cells [21]. Another study in glioma cells has shown that TIIA could inhibit viability of cells, their migratory potential and invasiveness, and decrease levels of Cyclin D1, Matrix metallopeptidase 9 (MMP-9) and Vimentin via regulation of miR-16-5p/Talin-1 axis [22].

Summary of studies that evaluated expression of miR-16-5p or its partners in cell lines is presented in Table 1.

**Table 1:** Expression of miR-16-5p or its partners in cell lines (∆: knock-down or deletion, TIIA: Tanshinone IIA, CuET: diethyldithiocarbamate-copper complex, EPCs: endothelial progenitor cells, RANKL: receptor activator of nuclear factor-κB ligand, IDH: Isocitrate dehydrogenase)

Tumor type	Targets/ Regulators and Signaling Pathways	Cell line	Function	Reference
Neuroblastoma	MYCN	SK-N‐BE(2), NB‐19, and SH‐EP Tet21N	↑↑ miR-16-5p: ↓ proliferation, migration, and invasion	[7]
	Circ-CUX1, DMRT2	HUVEC, GI-LI-N, SK-N-SH and IMR-32	∆ Circ-CUX1 (which suppresses miR-16-5p): ↓ proliferation, migration, invasion , and glycolysis	[8]
Osteosarcoma	Smad3	hFOB1.19, MG63, SaOS2, HOS, and U2OS	↑↑ miR-16-5p: ↓ proliferation, migration, invasion, and ↑ therapeutic effect of cisplatin	[9]
	TSPAN15, PI3K/AKT signaling pathway	hFOB 1.19, MG63, Saos2 and HOS	↑↑ miR-16-5p: ↓ viability, migration, invasion	[10]
	LINC00662, ITPR1	U2OS, SAOS-2, 143B, and MG63, HFOB 1.19	∆ LINC00662 (which sponges miR-16-5p): ↓ proliferation, migration, invasion , and stemness property maintenance	[23]
	hsa_circ_0005721, TEP1	hFOB, 143B, U-2OS, HOS and Saos-2	∆ hsa_circ_0005721 (which sponges miR-16-5p): ↓ viability, migration, invasion	[24]
Hepatocellular carcinoma	AGAP2-AS1, ANXA11, AKT signaling	LO2, Hep3B, HCCLM3, Huh7, MHCC-97 H and SMMC-7721	↑↑ AGAP2-AS1: ↑ proliferation, migration, invasion , and ↓ apoptosis	[11]
	IGF1R	SMMC-7721, HL-7702	↑↑ miR-16-5p: ↓ proliferation, migration, invasion, and EMT process	[12]
	TTN-AS1, cyclin E1, PTEN/Akt signaling pathway	Bel7404 and HepG2	∆ TTN-AS1 (which sponges miR-16-5p): ↓ sorafenib resistance, ↑ apoptosis ∆ miR-16-5p: ↑ sorafenib resistance, ↓ apoptosis	[13]
	SNHG22, EZH2, DNMT1	HLE-3, Huh7, HCCLM6, MHCC97H and SNU-398	∆ SNHG22 (which suppresses the transcription of miR-16-5p): ↓ proliferation, invasion, and angiogenesis ∆ miR-16-5p: ↑ proliferation, migration, invasion, and angiogenesis	[14]
Cervical cancer	CARM1	HeLa, C-33 A, CaSki, HeLa229, SiHa, END1/ E6E7	↑↑ miR-16-5p: ↓ colony formation, and radioresistance, ↑ apoptosis	[15]
	PDK4	HeLa, SiHa, HeLa/Dox, and SiHa/Dox	↑↑ miR-16-5p: ↓ proliferation, glucose consumption, lactate production, and ATP levels, and resistance to Dox treatment	[16]
	DLX6-AS1, ARPP19	End1/E6E7, SiHa, HeLa, C-33 A, and CaSki	∆ DLX6-AS1 (which sponges miR-16-5p): ↓ proliferation, migration, and EMT process, ↑ apoptosis	[25]
Breast cancer	AKT3, NF-κB pathway	BT-549 and MCF-7	↑↑ miR-16-5p: ↓ proliferation, migration, ↑ apoptosis, cell cycle arrest	[17]
	ANLN	MCF-7, T47D, MDA-MB-231, EMF-192 A, SKBR-3 and MCF-10 A, HEK293T	↑↑ miR-16-5p: ↓ proliferation, migration, invasion, and ↑ apoptosis, G2/M phase arrest	[18]
	VEGFA , Hypoxia-inducible factor-α (HIF-α)	MCF-7 and MDA-MB-231, MDA-MB-435, MDA-MB-468 and T47D, MCF10A	↑↑ miR-16-5p: ↓ proliferation, invasion, colony formation, ↑ apoptosis	[19]
	ATXN8OS, VASP	MCF-10 A MCF-7, and BT-549	∆ ATXN8OS (which sponges miR-16-5p): ↑ tamoxifen sensitivity	[20]
Gliomas	WEE1, CHEK1 and MCL1	A172, T98G, U251MG, U138MG and U87MG, TP365MG	↑↑ miR-16-5p: ↓ proliferation, viability, ↑ apoptosis, cell cycle arrest, response to irradiation and chemotherapy histone deacetylase inhibitor TS treatment: ↑ miR-16-5p ∆ HDAC3: ↑ miR-16-5p	[21]
	TLN1	T98G and A172	TIIA treatment: ↑ miR-16-5p ↑↑ miR-16-5p: ↓ proliferation, migration, invasion	[22]
Neuroendocrine tumors	SSTR2	INS-1 rat insulinoma cell line and GH3 rat pituitary GH- and PRL-producing cell line	octreotide treatment: ↑ miR-16-5p ↑↑ miR-16-5p: ↓ Proliferation, ↑ SSTR2 expression	[26]
Chordoma	Smad3	U-CH1 and U-CH2	↑↑ miR-16-5p: ↓ proliferation, migration, invasion	[27]
	LINC00662, RNF144B	U-CH1 and U-CH2	∆ LINC00662 (which sponges miR-16-5p): ↓ proliferation, migration, invasion, colony formation, and EMT process	[28]
Gastric cancer	PD-L1	PBMCs and CD3+ T cells AGS and NCI-N87	M1 Macrophage-Secreted Exosomes Carrying miR-16-5p: ↑ polarization of macrophages to its M1 phenotype, and T cell activation, ↓ PD-L1 expression	[29]
	Smad3	BSG823 and SGC-7901	Melatonin treatment: ↑ miR-16-5p: ↓ proliferation, ↑ apoptosis	[30]
	LINC00649, YAP1, Hippo signaling pathway	MGC-803 and SGC-7901	∆ LINC00649 (which sponges miR-16-5p): ↓ proliferation, migration, viability, ↑ apoptosis	[31]
	LINC00473, CCND2	BGC823, AGS, MKN-45, NCI-N87, GES-1	∆ LINC00473 (which sponges miR-16-5p): ↓ proliferation, migration, invasion, ↑ apoptosis, cell arrest	[32]
Lung cancer	WEE1	GLC-82 and HTB-182	Quercetin: ↑ miR-16-5p ↑↑ miR-16-5p: ↓ proliferation, colony formation, viability, ↑ apoptosis, and radiosensitivity	[33]
	Linc00210, PTK2	BEAS-2B, A549, Calu-3, H1299, SPCA-1, and PC-9	∆ Linc00210 (which sponges miR-16-5p): ↓ proliferation, invasion, ↑ apoptosis	[34]
	XIST, WEE1	H157, HCC827, A549 and H838	∆ XIST (which sponges miR-16-5p): ↓ colony formation, viability, ↑ apoptosis, and radiosensitivity	[35]
Colorectal cancer	PVT1, VEGFA, VEGFR1, AKT signaling	FHC, HCT116 and SW480, and HEK293T	∆ PVT1 (which sponges miR-16-5p): ↓ proliferation, migration, and invasion ↑↑ miR-16-5p: ↓ proliferation, migration, and invasion	[36]
	ALDH1A3, PKM2	HCT116, LoVo, DLD1 and RKO	CuET treatment: ↑ miR-16-5p and miR-15b-5p: ↓ glycolysis, viability and ↑ G2/M-phase arrest and apoptosis ∆ ALDH1A3 (a target of mir-16-5p): ↓ viability and clonogenicity	[37]
	ITGA2	Caco-2, SW480, SW620, LoVo, and HT29	↑↑ miR-16-5p: ↓ proliferation, migration, and invasion, ↑ apoptosis ∆ miR-16-5p: ↑ proliferation, migration, and invasion, ↓ apoptosis	[38]
Erythroleukemia		MEL cells	↑↑ miR-16-5p: ↑ erythroid differentiation of MEL cells by regulating ribosome biogenesis	[39]
Prostate cancer	AKT3		↑↑ miR-16-5p: ↓ cell survival, ↑ cell cycle distribution and apoptosis	[40]
	Cyclin D1/E1-pRb-E2F1 pathway	LNCaP	Ionizing radiation: ↑ miR-16-5p: ↓ proliferation, viability, and ↑ G0/G1 phase arrest	[41]
Chondrosarcomas	VEGF-A, PI3K/Akt signaling	JJ012, SW1353	Resistin treatment: ↓ miR-16-5p: ↑ VEGF-A-dependent EPCs angiogenesis	[42]
Giant cell tumor of bone		BMM cells	↑↑ miR-16-5p: ↓ RANKL-induced osteoclastogenesis	[43]
Papillary thyroid carcinoma	SNHG12	PTC cell lines	∆ SNHG12 (which sponges miR-16-5p): ↓ proliferation, migration and invasion and ↑ apoptosis	[44]
Renal cell carcinoma	PVT1	HK-2, A498, 786-O, ACHN and Caki-1	∆ PVT1 (which sponges miR-16-5p): ↓ proliferation, migration invasion, EMT process, and ↑ apoptosis ∆ miR-16-5p: ↑ proliferation, migration invasion, EMT process, and ↓ apoptosis	[45]
Bladder cancer	BIMP1/NFκB signaling pathway	T24 and 5637	↑↑ miR-16-5p: ↓ viability, ↑ autophagy and apoptosis	[46]
	LINC00649, JARID2	HCV-29, UMUC2, SW780, and T24	∆ LINC00649 (which sponges miR-16-5p): ↓ proliferation, migration, and invasion	[47]
Cholangiocarcinoma	R-2HG, ERα, YAP1	QBC939, HuCCT1, and HEK293T	IDH mutations: ↑ R-2HG production ↑ R-2HG: ↑ degradation of FTO so ↓ protein translation of the ERα: ↑ miR-16-5p: ↓ YAP1: ↓ proliferation and cell growth	[48]

### Animal studies

The tumor suppressor role of miR-16-5p has been verified in animal models of different types of cancers. Studies in these models have shown that over-expression of this miRNA or modulation of expression of lncRNAs that sponge this miRNA can block carcinogenic processes. For instance, transplantation of miR-15a‐, miR‐15b‐ and miR‐16‐expressing neuroblastoma cells into extremely immunodeficient mice has suppressed formation of tumors as well as expression of MYCN, suggesting that these miRNAs have a tumor suppressor role in neuroblastoma through targeting MYCN [7]. Another study in xenograft model of neuroblastoma has shown that knock down of the miR-16-5p-targeting circ-CUX1 leads to reduction of tumor growth [8].

In animal models of hepatocellular carcinoma, up-regulation of AGAP2-AS1 has enhanced tumor growth via down-regulating miR-16-5p [11]. Moreover, down-regulation of TTN-AS1 decreases tumor size and resistance to sorafenib through enhancement of expression of miR-16-5p [13]. In cervical cancer models, silencing of miR-16-5p target, PDK4 has enhanced efficacy of chemotherapy [16]. Moreover, silencing of DLX6-AS1 which targets miR-16-5p decreases tumor size [25]. Other studies in breast cancer, chordoma/chondrosarcoma, gastric cancer, lung cancer, colorectal cancer, bladder cancer and cholangiocarcinoma have confirmed a tumor suppressor role for miR-16-5p (Table 2).

**Table 2:** Function of miR-16-5p or its partners in animal models (∆: knock-down or deletion)

Tumor Type	Animal models	Results	Reference
Neuroblastoma	NOD.Cg-PrkdcscidIl2rgtm1Wjl/SzJ (NSG) mice	↑↑ miR-16-5p: ↓ bioluminescence, tumor size, and tumor weight	[7]
	BALB/c nude mice	∆ Circ-CUX1 (which suppresses miR-16-5p): ↓ tumor size, tumor weight, and tumor growth	[8]
Hepatocellular carcinoma	female BALB/c nude mice	↑↑ AGAP2-AS1: ↑ tumor growth and metastasis ∆ AGAP2-AS1: ↓ tumor growth and metastasis	[11]
	male Balb/c nude mice	∆ TTN-AS1 (which suppresses miR-16-5p): ↓ tumor size, tumor weight, sorafenib resistance	[13]
	male BALB/c nude mice	∆ SNHG22 (which suppresses the transcription of miR-16-5p): ↓ tumor growth and angiogenesis	[14]
Cervical cancer	BALB/c nude mice	∆ PDK4 (a target of miR-16-5p): ↑ chemotherapy efficiency	[16]
	BALB/c nude mice	∆ DLX6-AS1 (which sponges miR-16-5p): ↓ tumor sizes, volumes, and weights	[25]
Breast cancer	BALB/c nude mice	∆ mir-16-5p: ↑ tumor volume, proliferation and metastasis	[17]
	nude mice	↑↑ mir-16-5p: ↓ tumor growth	[19]
	BALB/c nude mice	∆ ATXN8OS (which sponges miR-16-5p): ↑ tamoxifen sensitivity	[20]
Chordoma	BALB/c athymic nude mice	↑↑ mir-16-5p: ↓ tumor volume and proliferation	[27]
	BALB/c nude mice	∆ LINC00662 (which sponges miR-16-5p): ↓ tumor volumes and tumor weight	[28]
Gastric cancer	BALB/c mice and NOD/SCID nude mice	M1 macrophage-secreted exosomes carrying miR-16-5p: ↓ tumor growth, volume and weight	[29]
	female BALB/c nude mice	∆ LINC00649 (which sponges miR-16-5p): ↓ tumor growth	[31]
	female BALB/c-nude mice	∆ LINC00649 (which sponges miR-16-5p): ↓ tumor growth, tumor weight proliferation, and metastasis	[32]
Lung cancer	nude mice	∆ LINC00649 (which sponges miR-16-5p): ↓ tumor growth, volume and weight	[34]
Colorectal cancer	male BALB/c nude mice	↑↑ mir-16-5p: ↓ tumor volume and weight	[36]
	male nude mice	CuET treatment: ↓ tumor volume and growth, ↑ apoptosis	[37]
	BALB/c nude mice	↑↑ mir-16-5p: ↓ tumor volume and growth	[38]
Chondrosarcoma	male nude mice	↑↑ Resistin: vessel markers VEGF-A and CD31, EPC markers CD34 and CD133, and vessel formation	[42]
Bladder cancer	male BALB/c nude mice	↑↑ miR-16-5p: ↓ tumor volume, weight, and growth	[46]
Cholangiocarcinoma	female nude mice	↑↑ R-2HG (which increases levels of miR-16-5p) : ↓ tumor growth	[48]

### Human studies

Down-regulation of miR-16-5p has been verified in clinical samples obtained from patients with different malignancies. Moreover, AGAP2-AS1 that decreases miR-16-5p levels has been shown to be up-regulated in hepatocellular carcinoma tissues, particularly in metastatic and recurrent ones. In addition, expression levels of AGAP2-AS1 and miR-16-5p have been correlated with clinical parameters and poor prognosis of patients with this type of cancer [11]. In neuroblastoma, up-regulation in circ-CUX1 that sponges miR-16-5p has been correlated with advanced TNM stage, low differentiation grade and lymph node metastasis [8]. In breast cancer patients, miR-16-5p has been shown to have low expression. Notably, patients with low expression of miR-16-5p have been found to have a lower survival rate compared with those having high expression of miR-16-5p [17].

In the majority of CLL cases, miR-15a and miR-16-1 have been shown to be lost or down-regulated [6]. Moreover, assessment of GO database has led to identification of enrichment of MCL1 Apoptosis Regulator, BCL2 Family Member (MCL1), B-cell lymphoma 2 (BCL2), ETS Proto-Oncogene 1 (ETS1), or Jun Proto-Oncogene, AP-1 Transcription Factor Subunit (JUN) in miR-16 signature. Notably, these genes are involved in the regulation of apoptosis and cell cycle [6].

Several studies have reported down-regulation of this miRNA in nearly all examined malignant tissues except for ovarian cancer tissues. Similarly, lncRNAs or circRNAs that decrease expression of miR-16-5p have been found to be up-regulated in cancer samples compared with non-cancerous controls (Table 3).

**Table 3:** Dysregulation of miR-16-5p or its partners in clinical samples (NB: Neuroblastoma, FAM: fetal adrenal medulla, ANCTs: adjacent non-cancerous tissues, OS: Overall survival, TNM: tumor-node‐metastasis, ccRCC: clear cell renal cell carcinoma)

Tumor type	samples	Expression of miR-16-5p or other genes (Tumor vs. Normal)	Kaplan-Meier analysis (impact of miR-16-5p dysregulation)	Association of expression of miR-16-5p or expression of other genes with clinicopathologic characteristics	Method by which RNA was detected	Reference
Neuroblastoma	R2 database, containing 105 NB patients	Down				[7]
	50 pairs of tumor tissues and FAM tissues	Upregulation in circ-CUX1 (which sponges miR-16-5p)	Lower OS	Upregulation in circ-CUX1 was correlated with advanced TNM stage, low differentiation grade and lymph node metastasis.		[8]
Osteosarcoma	40 pairs of tumor tissues and ANCTs	Down	Lower OS		SYBR® Premix Ex TaqTM Kit	[9]
	51 pairs of tumor tissues and ANCTs	Upregulation in LINC00662 (which sponges miR-16-5p)	Lower OS	Upregulation in LINC00662 was correlated with distant metastasis, TNM stage, and tumor size.		[23]
	30 pairs of tumor tissues and ANCTs	Upregulation in hsa_circ_0005721 (which sponges miR-16-5p)			SYBR®Premix Ex Taq™	[24]
Hepatocellular carcinoma	137 pairs of tumor tissues and ANCTs	Upregulation in AGAP2-AS1 (which sponges miR-16-5p) Downregulation in miR-16-5p		Upregulation in LINC00662 was correlated with large tumor size, metastasis, recurrence and high histological grade tissues	-	[11]
	100 pairs of tumor tissues and ANCTs	Downregulation in miR-16-5p			SYBR PrimeScriptTM RT-PCR Kit	[12]
	60 pairs of tumor tissues and ANCTs	Upregulation in SNHG22 (which suppresses transcription of miR-16-5p)	Lower OS		SYBR Green PCR kit	[14]
Cervical cancer	63 pairs of tumor tissues and ANCTs	Upregulation in CARM1 (a target of miR-16-5p) Downregulation in miR-16-5p		Upregulation in CARM1 was correlated with higher clinical staging and poorer tumor differentiation	SYBR Green PCR kit	[15]
Gliomas	72 pairs of tumor tissues and ANCTs	Downregulation in miR-16-5p			SYBR Green I fluorescence method	[17]
	GEO and TCGA databases	Upregulation in ANLN (a target of miR-16-5p)	Lower OS			[18]
	40 pairs of tumor tissues and ANCTs	Downregulation in miR-16-5p			SYBR Green kit	[19]
	22 pairs of tumor tissues and ANCTs	Upregulation in ATXN8OS (which sponges miR-16-5p)			SYBR® Premix Ex TaqTM reagent	[20]
Gliomas	79 patients with astrocytic gliomas and 9 non-neoplastic brain samples	Downregulation in miR-16-5p			TaqMan probe	[21]
Chordoma	12 chordoma tissues and 12 nucleus pulposus tissues 10 chordoma tissues and 5 nucleus pulposus tissues	Downregulation in miR-16-5p			SYBR-Green PCR Master Mix	[27]
	30 pairs of tumor tissues and ANCTs	Upregulation in LINC00662 (which sponges miR-16-5p)			RT2 SYBR Green FAST Mastermix or miScript SYBR Green PCR Kit	[28]
Gastric cancer	54 pairs of tumor tissues and ANCTs TCGA dataset	Downregulation in miR-16-5p			One-Step TB Green TM PrimeScript TM RT-PCR kit	[31]
	53 pairs of tumor tissues and ANCTs	Upregulation in Linc00210 (which sponges miR-16-5p)		Upregulation in LINC00473 was correlated with a higher risk of lymphatic metastasis, a higher incidence of vascular cancer embolus, and advanced TNM stage.	TB Premix Ex Taq	[32]
Lung cancer	40 pairs of tumor tissues and ANCTs	Upregulation in Linc00210 (which sponges miR-16-5p)			SYBR Premix Ex Taq II and Perfect Real Time	[34]
	31 pairs of tumor tissues and ANCTs	Upregulation in XIST (which sponges miR-16-5p)			SYBR Green Master Mix	[35]
Colorectal cancer	72 pairs of tumor tissues and ANCTs	Upregulation in PVT1 (which sponges miR-16-5p)	Lower OS	Upregulation in PVT1 was significantly correlated with lymph node metastasis, distant metastasis, and TNM (tumor, node, metastasis) stage	SYBR Green	[36]
	42 pairs of tumor tissues and ANCTs	Upregulation in ALDH1A3 (a target of miR-16-5p)	Lower OS			[37]
	GEO database: GSE75970, GSE74602, GSE89076, and GSE10950	Upregulation in ITGA2 (a target of miR-16-5p)				[38]
Chronic lymphocytic leukemia	224 CLL cases and 224 matched controls	miR-16-5p levels were unrelated to CLL risk.			TaqMan probes	[49]
Chondrosarcoma	9 human chondrosarcoma tissues and 9 normal cartilage	Downregulation in miR-16-5p				[42]
Giant cell tumor of bone	17 GCT tissue and 4 cancellous bone as controls	Downregulation in miR-16-5p			iTaq™ Universal SYBR Green Supermix	[43]
Ovarian cancer	142 ovarian cancer patients, and 97 healthy controls	Upregulation in miR-16-5p	No correlation between the gene expression levels, and the survival time			[50]
Renal cell carcinoma	25 patients with ccRCC	Upregulation in PVT1 (which sponges miR-16-5p)		Upregulation in PVT1 was correlated with TNM stage, Fuhrman grade, lymph node metastasis and tumor size.	SYBR Green	[45]

## Discussion

miR-16-5p is an example of miRNAs with tumor suppressor role in almost all assessed tissues. This speculation is based on the observed down-regulation of this miRNA in nearly all examined malignant tissues except for ovarian cancer tissues. Moreover, a number of studies have reported up-regulation of lncRNAs that target this miRNA or specific targets of this miRNA. This miRNA has been found to be sponged by some lncRNAs and circRNAs, namely LINC00662, LINC00649, LINC00473, LINC00210, PVT1, XIST, AGAP2-AS1, DLX6-AS1, TTN-AS1, circ-CUX1 and hsa_circ_0005721. These observations indicate the complexity of the network through which miR-16-5p exerts its tumor suppressor effects. Moreover, abnormal up-regulation of the mentioned lncRNAs and circRNAs is regarded as a possible mechanism for down-regulation of miR-16-5p along with genomic variations in the genetic locus of this miRNA.

Phosphoinositide 3-kinase (PI3K)/AKT, Phosphatase and tensin homolog (PTEN)/AKT, NF-κB, Hippo and E1-pRb-E2F1 pathways are among signaling pathways being affected by dysregulation of miR-16-5p. Thus, down-regulation of miR-16-5p can lead to over-activity of cancer-related signals enhancing cell survival.

Down-regulation of miR-16-5p or up-regulation of lncRNAs/circRNAs that sponge this miRNA has been shown to be associated with malignant features of different cancers such as neuroblastoma, osteosarcoma, renal cell carcinoma and colorectal cancer, indicating a role for miR-16-5p as a prognostic marker in human cancers. In fact, down-regulation of this miRNA has been detected in samples with low level of differentiation and high propensity to local and distant metastases. Thus, patient with low levels of expression of this miRNA has exhibited poor clinical outcomes.

Since this miRNA can be detected in the peripheral blood, it represents a novel non-invasive strategy for early detection of cancer. However, since it is down-regulated in several types of cancers, the type of cancer cannot be detected through this route. Moreover, evaluation of levels of miR-16-5p in cancer patients can be used for follow-up after removal of primary tumor.

The mechanism behind down-regulation of miR-16-5p in malignant tissues is not investigated thoroughly, although deletion in the genomic region coding this miRNA is a putative mechanism. Moreover, up-regulation of lncRNAs/circRNAs that sponge this miRNA is a well-established mechanism for its down-regulation in different cancers. Induction of DNA methylation in miR-16-5p is another mechanism of down-regulation of this miRNA in cancers [14]. Future studies are needed to find possible epigenetic alterations that affect transcription of precursor of miR-16-5p.

Different studies have shown the effects of miR-16-5p in regulation of chemosensitivity, radiosensitivity as well as response to the targeted therapy by sorafenib. From a clinical point of view, up-regulation of miR-16-5p is a potentially effective modality for suppression of tumor growth and defeating chemotherapy resistance. However, introduction of miR-16-5p mimic into cancerous cells needs a specific strategy to shield the miRNA mimics from self-hydrolysis or degradation by RNases. Without these considerations, the short half-life of naked RNA mimics reduces the potential effects of miRNAs [51]. Moreover, issues regarding the toxicity or nonspecific cell-targeting nature of miRNA carriers should be solved. These issues have attenuated the pace of entering miRNA mimics into the clinical setting.

Cumulatively, miR-16-5p is a putative tumor suppressor miRNA that can be used as a therapeutic modality in different cancers. However, the biosafety and bioavailability issues should be solved before introduction of this modality in clinical settings.

## Data Availability

The analyzed data sets generated during the study are available from the corresponding author on reasonable request.

## Declarations

**Consent of publication**.
